# Regional integration in the EU and ASEAN in the period of declining multilateralism and corona shocks

**DOI:** 10.1007/s10368-020-00480-4

**Published:** 2020-08-01

**Authors:** Suthiphand Chirathivat, Natthanan Kunnamas, Paul JJ Welfens

**Affiliations:** 1 ASEAN Studies Center, Chulalongkorn University, Bangkok, Thailand; 2 Faculty of Political Science, Chulalongkorn University, Bangkok, Thailand; 3 European Institute for International Economic Relations (EIIW) / University of Wuppertal, Wuppertal, Germany; 4 AICGS/Johns Hopkins University, Washington, DC USA

## Introduction

Regional integration has been a key part of economic globalization since 1957; at first in the form of European integration, later also in Asia (with the Association of Southeast Asian Nations - ASEAN), Latin America (e.g., Mercosur) and Africa (e.g., ECOWAS). As a customs union, the European Union (EU) is – in terms of its institutional framework - most similar to Mercosur, but in economic terms there is a considerable similarity to the ASEAN countries which have decided to start an ASEAN single market in 2019. EU integration has been a successful long-term project over many decades – the number of member countries increased over half a century from the initial grouping of six countries to 28 countries, but 2016 became a historical watershed year as the United Kingdom held a referendum on continued EU membership and a narrow majority voted in favor of leaving the EU, much to the surprise of most British experts, the European Commission and the European Parliament - as well as the German and French governments. For the first time in the history of EU integration, a majority within a member country had refused continued EU membership and this, in a large European economy, is a shock event not just for the European Union but also raises new questions in other regional integration areas.

ASEAN is a rather dynamic integration group which has partly been inspired by the integration history in Europe on the one hand, but on the other hand there has also been growing economic linkages between the EU and ASEAN. While it is true that ASEAN currently has less institutional integration than the European Union, the growing group of ASEAN countries also stands for a regional integration success story and its ability to initiate is own regional single market – with the same four freedoms as in the EU’s single market project starting in 1993 – has been remarkable. To what extent ASEAN will be able to maintain a low profile institutional setting remains to be seen in the new world economy with US anti-multilateralism and rising US-China economic and political rivalry.

While the rising number of integration projects in the world economy and an increase in the number of countries active in regional integration schemes have suggested that regional integration is a strong pillar of both economic integration, globalization and multilateralism, 2016 became a critical historical watershed marker for another reason: The rather surprising majority of Donald Trump in the US presidential elections that year is the fifth case – out of the 45 presidents of the United States thus far - where the label of populism seems to be appropriate and this populist president declared during the election campaign in 2016 that he favored bilateralism over multilateralism, i.e., the international governance structures based largely on internationally accepted rules and international organizations. There is a natural tension between regional integration schemes, which themselves use complex treaties involving many countries at the same time, and the bilateralist approach which typically favors privileged interaction amongst large economies and also an undermining of global international organizations.

Under President Trump, the World Trade Organization is an obvious target of the anti-multilateralism pervasive in the White House. In the period of such complex international changes, the coronavirus shocks – namely both a public health and an economic shock – in particular have hit the world economy in 2020 and pose new challenges for regional integration clubs, such as the EU and ASEAN (and others), and the wider global economy. The following contributions look into EU and ASEAN economic and political dynamics – with a deliberate interdisciplinary focus (from economists, a historian and political scientists) in this special issue. Most papers presented here have been extensively discussed at workshops at both Chulalongkorn University in Bangkok in 2018 and at an international conference which was hosted by Chulalongkorn University, the EIIW/University of Wuppertal and the European Commission in Brussels in 2019.

The first paper is by Paul JJ Welfens and takes a look at “Trump’s Trade Policy, BREXIT, Corona Dynamics, EU Crisis and Declining Multilateralism”. This broad and deep analysis of integration developments in the EU, contradictory US trade policy and the unfolding of the corona shocks in Asia, Europe and the US (and the world economy) show a critical overlap of international economic challenges; including critical points of post-BREXIT EU integration dynamics where serious risks in terms of a potential new Euro Crisis are identified. The newly developed formula of optimum import tariffs in open economies with outward foreign direct investment gives crucial insights on the non-optimality of both US and UK tariff policy under populist political leaders. As regards the risk of new Euro Crisis, and potential further disintegration of the EU, critical questions are raised with respect to EU and EU member countries’ fiscal policies and the lack of institutional reforms. The weakening of multilateralism by the Trump Administration has dealt a serious blow to the global set of rules and the question arises as to what political pattern possibly could replace the historical US leadership over the 1944–2016 period. One new option could be a networked leadership shared between regional integration clubs.

Andrew Crozier raises key topics and issues of British policy in a broader historical context and thus can also give the reader a broader perspective on BREXIT. His contribution “British Exceptionalism: Pride and Prejudice and Brexit” puts a focus back on the period when it developed its classic form in the wake of the Napoleonic Wars. During the nineteenth century, the British state concentrated on the creation of a global empire and by the 1890s was in a state of ‘splendid isolation’ with respect to Europe. Simultaneously, the rise of Germany meant that Britain could not ignore Europe entirely. During the first half of the twentieth century, Britain became involved in two major wars, which to a considerable extent revolved around the need to curb German power. After the First World War, Britain wanted to focus once again upon her global interests and this was reflected in her reluctance to embrace integrationist initiatives such as the Briand Plan. Although the Second World War destroyed the basis of Britain’s Empire, the feeling that British superiority had saved the world only reinforced the sense of exceptionalism by adding to it a sense of ‘pride’. After 1945, British power steadily waned and the British state increasingly came under pressure from Washington to join the European Economic Community. The potential of Germany to dominate this grouping and British apprehension of such a development led to ‘prejudice’ in respect of Germany which later translated itself into prejudice against Europe as a whole. Once inside the European Community, Britain accordingly became somewhat of an awkward partner. Never entirely comfortable within the EU, a secessionist movement grew which ultimately forced the referendum of 2016, the result of which came as a surprise to many observers in Europe and beyond. It is rather unclear whether or not the United Kingdom can easily be stabilized in economic and political terms post-BREXIT.

Rolf J. Langhammer and Suthiphand Chirathivat discuss “ASEAN and the EU Challenged by “Divide and Rule” Strategies of the US and China: Evidence and Possible Reactions”. The United States and China, both global superpowers, have launched attacks upon the coherence of ASEAN and the EU by offering individual member states privileges if they depart from common policies of the two integration schemes. Key motives behind those offers and the ways they are addressed to the member states are reflected upon by the authors who consider such “divide and rule” policies as serious challenges to the collective bargaining power of ASEAN and the EU, respectively. Moreover, strategies to counter these challenges are discussed. For both China and the US, the authors see economic and political targets as the key elements of motivation. Economically, each of the two countries wants to gain superiority in paving freeways for their suppliers of technology, goods and services to the markets of the two schemes against the competitive pressure of the other country. Politically, the two integration schemes have become contested areas in the geopolitical struggles between the two countries. For the EU, as the more advanced scheme of deep integration, the paper recommends a closer convergence between EU policies and the demand of the electorate, to prefer more cooperation projects over deeper integration steps, and to motivate the private sector, in particular higher FDI inflows and options to include foreign investors, in order to stand up against “divide and rule” strategies. Strengthening ASEAN integration through the removal of non-tariff barriers to trade within ASEAN is crucial.

Ajaree Tavornmas and Kasira Cheeppensook consider topics and issues of international ocean governance with respect to links between the EU and Thailand/ASEAN. The authors’ contribution is titled “Shaping Ocean Governance: A Study of EU Normative Power on Thailand’s Sustainable Fisheries”. The EU has long been championing an agenda for better ocean governance based on a cross-sectoral, rules-based, international approach and indicated its role as a strong global actor in this field early on. The European Union, as reflected through the strategies it has adopted during the last decade (2005–2015), aims to shape international ocean governance on the basis of its experience in developing a sustainable and ethical approach to ocean management, notably through its environment policy and regulatory regime. This paper observes a significant transition of EU internal policy towards a more externally-oriented one, as well as its ambition in exporting EU norms to third countries. It seems that the EU aims to lead this maritime and fisheries domain as a global actor, diffusing norms via interstate relations. The case study of EU policy towards Thailand’s fisheries policy, resulting in Thailand’s adoption of a sustainable fisheries policy in 2015, is explored in this research paper. In addition, the paper aims to analyze the development and evolution of Thailand’s sustainable fisheries policy during 2015–2019 and to examine the rationale behind Thailand’s shift towards a more environmentally- and socially-friendly fisheries policy. It is quite important to understand how the EU tries to export its standards (as an EU28 country group) to other parts of the world economy. Ocean governance could become increasingly important over time.

Infrastructure is a new key field of global economic rivalry and indeed it has also become an important topic for regional economic development. China, Japan and the EU have become active here in the global economy, but their strategies are rather different as emphasized by Werner Pascha who highlights analytically these crucial developments in his paper “The Quest for Infrastructure Development from a “market creation” perspective: China’s “Belt and Road”, Japan’s “Quality Infrastructure” and the EU’s “Connecting Europe and Asia””**.** Pascha puts the focus on some key analytical points related to the public good aspects of modern infrastructure – an analysis which has been partly neglected in the Economics literature on infrastructure investment projects. The paper deals with the interplay of major international infrastructure initiatives, in particular China’s Belt and Road Initiative, Japan’s Partnership for Quality Infrastructure and the EU’s strategy on “Connecting Europe and Asia”. Their co-evolution is interpreted as the creation and further development of a new market, whose characteristics, like its complexity, its properties as an international public good and its oligopolistic supply structure, create interesting insights. The paper finds that the initiatives have adjusted to each other, in line with expectations from a market perspective. While China’s initiative at first followed a “low-price” strategy, Japan reacted with a “quality infrastructure” approach, also winning support from multilateral fora such as the G7 and G20. Gaining a deeper insight into the international contest between China, Japan and the EU is crucial to understanding international economic relations and some limitations to international cooperation stemming from new rivalries.

Evelyn S. Devadason and Shujaat Mubarik consider the links between the European Union and ASEAN in the field of trade. The title of their paper is “ASEAN and the EU: An Assessment of Interregional Trade Potentials”. Though negotiations on an Association of Southeast Asian Nations – European Union (ASEAN-EU) trade agreement began in 2007, the region-wide agreement stalled, and the EU has since pursued bilateral agreements with individual ASEAN member states (AMS). Nevertheless, the goal of forming an interregional agreement remains as the European Commission and the AMS are currently participating in a stocktaking exercise to explore the prospects towards the resumption of region-to-region negotiations. In this paper, a stochastic frontier specification of the gravity model is employed to identify and compare the performance (efficiency) of exports relative to the maximum export levels for the ASEAN-EU partnership. The findings, based on a panel dataset of two-way bilateral exports between the ASEAN10 and the EU28 over the 2000–2016 period, indicate a low-level of export efficiency. It seems that there is considerable room for higher EU-ASEAN trade in the future, possibly with digital services becoming a new growing field in the long run. With BREXIT, the smaller EU27 will be quite interested in both maintaining and expanding trade with ASEAN, but the UK in turn will also be quite eager to witness UK-ASEAN trade creation in the context of new FTA treaties to be concluded after January 31st 2020.

Economic analysis is often useful for understanding key elements of regional integration and inter-regional cooperation, but in many cases a political science perspective is an indispensable corner stone for a broader understanding of complex international dynamics. Julie Gilson’s contribution “EU-ASEAN Relations in the 2020s Pragmatic Inter-Regionalism?” indeed sheds new light on the relevant issues. In the late 1990s and early 2000s, the world witnessed a proliferation of region-to-region institutional frameworks. There was a recognition that scale and leverage could create an advantage for economic relations, that security could benefit from cross-regional dialogue and initiatives, and that some of the many global challenges, from global climate change to resource depletion, could be addressed more effectively at regional and even inter-regional levels. The EU-ASEAN dialogue itself presents an important model for inter-regional cooperation at the heart of these tangled institutional webs. This analytical perspective assesses the ways in which the changing multilateral landscape and intra-regional crises within ASEAN and the EU have altered the relevance of inter-regional dialogue and initiatives. It becomes clear that an innovative EU27 international cooperation approach should take a broader approach than the current, rather slow, EU-ASEAN cooperation.

China’s new military strength has become visible in many fields – certainly in the South China Sea region with localized conflicts involving various countries. Kasira Cheeppensook takes a fresh look at the issues. She analyzes “ASEAN in the South China Sea conflict, 2012–2018: A lesson in conflict transformation from normative power Europe”. For decades, overlapping territorial claims in the South China Sea have had a destabilizing effect in East and Southeast Asia, with broader implications beyond the region. Four ASEAN countries (Brunei, Malaysia, the Philippines, and Vietnam) are direct claimants in the South China Sea conflict. ASEAN’s role, as a regional organization, in facilitating peaceful resolution of these claims and maintaining stability is challenging because the conflict presents potentially divisive rifts amongst ASEAN members themselves. This paper explores ASEAN’s role in managing the South China Sea conflict by examining the actions of two non-claimant states which functioned as country coordinators for ASEAN–China relations from 2012 to 2018: Thailand and Singapore. The efforts of these two countries as honest political brokers shed new light on how ASEAN can deal with this ongoing crisis so as to ensure the organization’s ongoing effectiveness and sustain regional harmony. Under the Normative Power Europe framework, the roles of non-claimants are explored in the regional conflict transformation process.

The EU integration club has for many years tried to exert normative power in various regions of the world. Natthanan Kunnamas’ contribution “Normative Power Europe, ASEAN and Thailand” takes a closer look at both the EU and ASEAN in general and in particular at the role of Thailand in this important context. Recognizing the state of political, democratic, and humanitarian problems in ASEAN, as well as the existence of a stumbling block for the two regional organizations’ relations - regarding human rights, the European Union has opted for a more pragmatic approach in two ways: firstly, by addressing a wide range of developmental issues and agendas in ASEAN political, economic, and socio-cultural cooperation; secondly, by implementing an adaptative policy to focus more on a region-to-state approach through development aid packages, and by fostering agreements of cooperation, especially with Thailand, a country case study. With different levels of leverage possessed by the EU on the region-to-region versus region-to-state platform, it has become clear that the EU’s normative pressure directed specifically toward Thailand yields a more desirable result than what it achieves with respect to ASEAN. The pressure by the EU to make Thailand conform to a set of values, i.e., liberty, democratic elections, freedom of expression, just and fair trade has, at times, generated certain challenges in the relations between the two parties, particularly during the period of military junta rule between May 2014 and July 2019. The analysis, therefore, evaluates whether bilateral relations between the EU and Thailand contribute to the EU’s normative aims using Ian Manners’ Normative Power Europe (NPE) concept to assess the extent to which the European Commission could exert normative pressures contributing to changes in Thailand through four examples, namely calls for *vox populi* elections and the protection of human rights, the campaign for the abolition of capital punishment, a ban on illegal fishing, and the implementation of EU-style emission trading schemes (ETS). ETS has become an increasingly important field of both national economic policy and climate policies with its global dimension and thus deserves particular attention.

There is a long history of EU-ASEAN cooperation, at least in the decades after the EU had effectively concluded trade integration. Marissa Maricosa Acierto Paderon takes a closer look at cooperation dynamics and perspectives under the heading “Opportunities in ASEAN-EU Economic Cooperation”. The European Union has been a dialogue partner of the Association of Southeast Asian Nations since 1977. This dialogue partnership was institutionalized with the signing of the ASEAN-EEC Cooperation Agreement on 7 March 1980. Since that agreement, EU-ASEAN dialogue relations have grown and expanded in both scope and depth. From the limited issues of trade, investment and development cooperation, its partnership has extended to social and cultural affairs; and political and security dialogue. The findings indicate that the ASEAN and EU are ‘natural trading partners’ and should pursue a region-to-region FTA. Institutional diversities in the ASEAN region as well as the BREXIT stress-test for the EU27 and Europe, respectively, have created a complex international setting for the near future.

With the overlap of regional dynamics, the US-China conflict and the corona shock affecting the world economy, adjustment options have narrowed at least for a few years and in both the EU and ASEAN – as well as in other integration areas – new reflections will emerge about the economic and political benefits, but also risks, of regional integration and networked international cooperation. While many voters in certain democracies – hardest hit by the public health and economic shocks of the coronavirus pandemic – might translate the shocks into long-term political frustration, there is also new economic pressure for more international benchmarking as certain countries have coped relatively successfully with the corona shocks and previous economic or political shocks. ASEAN countries show a rather favorable record in 2020 – compared to parts of the EU – which might be considered as a signal that a large share of the institutional capital in the region is working and valuable where the accumulation of said capital has, of course, taken place over decades. One cannot overlook that the US-Sino trade conflicts will raise new questions in Europe and Asia. We greatly appreciate the support of the the European Institute for International Economic Relations (EIIW/University of Wuppertal) and Chulalongkorn University in relation to the two workshops; the hospitality of the European Commission in Brussels is also much appreciated. Finally, the organizational and editorial support of our staff in Bangkok and Wuppertal is also recognized. European-Asian intellectual cooperation will hopefully continue on an even broader basis in the future and the interdisciplinary overlap of the fields of Economics, History and Political Science should be fruitful for understanding new critical future challenges as well.

Bangkok and Wuppertal, June 25, 2020

Suthiphand Chirathivat, Natthanan Kunnamas and Paul JJ Welfens

